# Facial Paresthesia, a Rare Manifestation of Hereditary Neuropathy With Liability to Pressure Palsies: A Case Report

**DOI:** 10.3389/fneur.2021.726437

**Published:** 2021-11-16

**Authors:** Lisa De Kock, Fréderic Van der Cruyssen, Leonore Gruijthuijsen, Constantinus Politis

**Affiliations:** ^1^Faculty of Medicine, University Leuven and Maxillofacial Surgery Department, University Hospitals Leuven, Leuven, Belgium; ^2^OMFS IMPATH Research Group, Department of Imaging and Pathology, Faculty of Medicine, University of Leuven and Oral and Maxillofacial Surgery, University Hospitals Leuven, Leuven, Belgium

**Keywords:** paresthesia, hereditary neuropathy with liability to pressure palsy, trigeminal neuropathy, PMP22 deletion, case report

## Abstract

Trigeminal sensory neuropathy can be caused by a variety of conditions, including local, traumatic, iatrogenic, or systemic causes. Diagnosis and management remain a challenge for maxillofacial surgeons and neurologists. Therefore, a good clinical examination and objective tests and imaging are needed when diagnosing patients who present with facial numbness. We present a case with spontaneous episodes of facial paresthesia. He was diagnosed with hereditary neuropathy with liability to pressure palsies (HNPP), a rare condition that affects the peripheral nerves. Only a few case reports that describe involvement of the cranial nerves in patients with HNPP were found in the literature, and facial paresthesia has not been previously reported.

## Introduction

According to the International Association for the study of Pain (IASP) terminology (1), paresthesia is defined as an abnormal sensation, whether spontaneous or provoked. It is not considered unpleasant, in contrast to dysesthesia, which is preferentially used to describe an abnormal sensation that is considered unpleasant. Paresthesia is frequently encountered in clinical practice by maxillofacial surgeons and neurologists, though diagnosis and management can be challenging. The condition has a broad differential diagnosis, which often makes it difficult to find the cause. Local, traumatic, or iatrogenic factors are the most common causes of paresthesia in the maxillofacial region, and systemic causes are rare (2). We report the case of a patient who presented with spontaneous episodes of facial paresthesia and was diagnosed with hereditary neuropathy with liability to pressure palsies (HNPP). The involvement of cranial nerves has seldom been described in case studies of HNPP. The purpose of this case report is to review this rare presentation and to examine the process for differential diagnosis in a patient with spontaneous paresthesia in the distribution area of the trigeminal nerve.

## Case Description

A 25-year-old Afro-American male was referred to the Department of Oral and Maxillofacial Surgery by his family doctor because of recurrent episodes of facial paresthesia. These episodes spontaneously started 3 months prior and were observed for the first time after severe alcohol consumption. They could develop any time during the day, but mostly occurred during meals and long conversations. The paresthesia was reported bilaterally in the distribution area of the mandibular nerve, particularly the mental and auriculotemporal nerve. No pain or hypoesthesia was described. He did not have recent dental treatment. These complaints impeded his daily life and professional activities due to concentration problems. A few weeks earlier, he experienced hypoesthesia in the left little finger, left foot, and the medial side of the left wrist for approximately 1 week. These symptoms were often encountered in the past, but never lasted as long as 1 week. The family doctor prescribed corticosteroids, but this did not improve the symptoms.

The patient's medical history included heterozygote sickle cell trait (HbAS) and alpha-thalassemia minor, for which he was scheduled for regular routine follow-ups with his hematologist. He had a history of smoking (2 pack-years). He had experienced tinnitus of unknown etiology for more than 4 years. In addition, the patient followed up regularly with a cardiologist for a second-degree atrioventricular block type 1. At 21 years old, he was diagnosed with HNPP. Genetic analysis of *PMP22* confirmed deletion of the chromosome 17p11.2 region. Electromyography (EMG) of the upper and lower limbs showed multifocal demyelinating anomalies with diminished sensory and motor conduction velocity. In this regard, he occasionally encountered transient episodes of muscle weakness and hypoesthesia in the arms and legs, as mentioned earlier.

**Figure 1:**
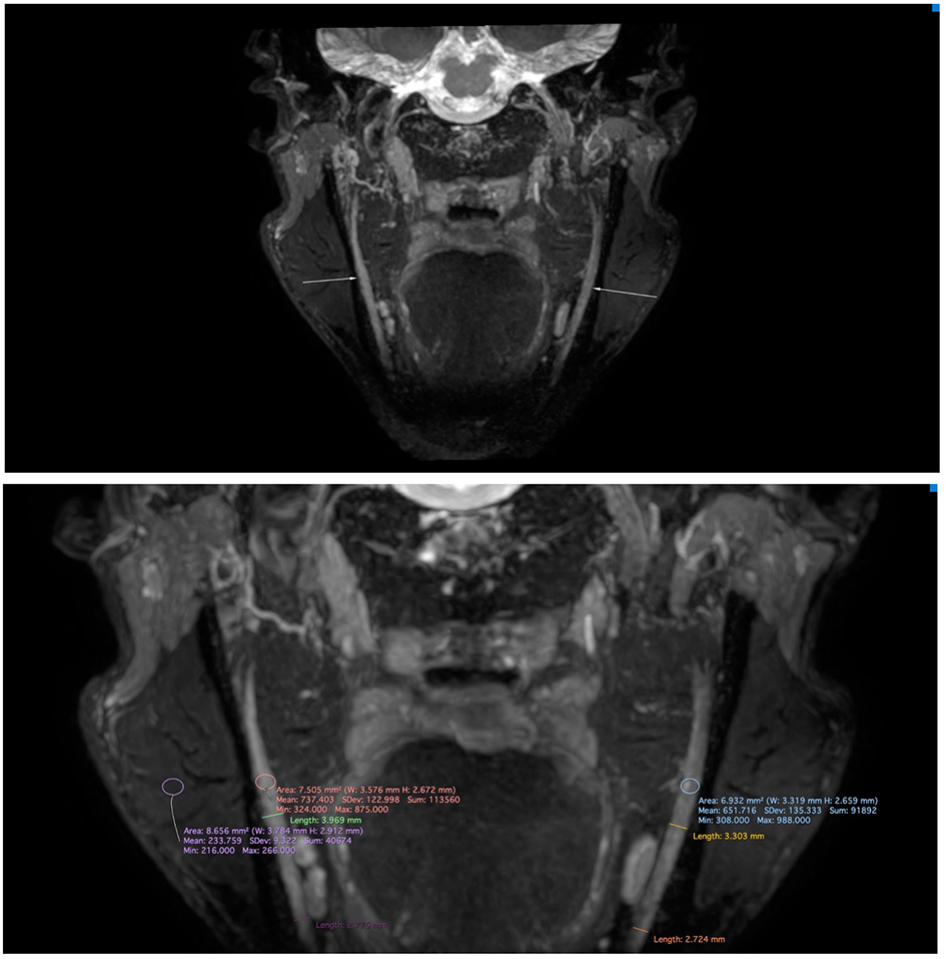
Magnetic resonance neurography of the mandibular nerve on a 3T Philips Ingenia with standard 32 channel head coil (Philips, Best, the Netherlands) applying a 3D CRANI sequence (4). The image was reconstructed using maximum intensity projection and multiplanar reformatting. Note the slightly increased nerve calibers and signal intensities, which are more pronounced on the right inferior alveolar nerve.

Upon clinical examination, the trigeminal and facial nerves were normal. Symptoms could not be elicited. There were no clinical signs of a disorder in the temporo-mandibular joint, and further clinical examination was normal. A panoramic radiograph and lateral head film showed no aberrancies except horizontally impacted wisdom teeth. Blood analysis showed microcytic hypochromic anemia, which can be attributed to the alpha-thalassemia minor. He had no vitamin deficiencies. HIV infection, hepatitis B, and syphilis were excluded by appropriate tests. Before our exam, he had been assessed by the neurology department. This showed a symmetric motor function of the facial nerve, normal sensibility of the trigeminal nerve and a normal examination of the remaining cranial nerves. Strength testing revealed a reduced flexion strength of the left little finger and reduced extension strength of the left great toe. On the left side the little finger and medial side of the hand as well as the upper side of the left foot showed a diminished light touch and pinprick sensibility. Vibration perception of the left hand and foot was normal. The right side was completely unremarkable. Coordination of a heel-shin slide and finger-to-toe test was normal. The reflexes were symmetric. An EMG of the left ulnar and median nerve did not yield pathological findings other than the known HNPP. Blink testing of the facial muscles were within normal limits. Quantitative sensory testing (QST) according to the DFNS protocol (3) revealed thermal sensory disturbances in the trigeminal distribution. On the right side, the cold pain threshold was increased, and the warmth detection threshold was clearly lower (Table 1). Magnetic resonance neurography (MRN) of the trigeminal nerve showed slightly increased nerve calibers and signal intensities, more pronounced on the right inferior alveolar nerve (IAN) (Figure 1). Intramuscular injections with vitamin B complex once a week were prescribed. After 6 weeks the patient reported improvement of his symptoms. The episodes of facial paresthesia were decreased in number and intensity and did no longer impeded his daily life activities. Vitamin B supplements were interrupted without recurrence or exacerbation of symptoms.

**Table 1:** Quantitative sensory testing according to the DFNS protocol.

**Variable**		
VAS pain score	0/10	
Allodynia	–	
Hyperpathia	–	
Hyperalgesia	–	
Hypoesthesia	–	
Paresthesia	+	
Affected dermatome, %	0	
Directional sense	10/10	
Stimulus localization	5/5	
Subjective score	10/10	
	**Left**	**Right**
Mechanical pain threshold (Von Frey filaments), mN	0.08	0.08
Tactile threshold, mN	64	64
Two-point discrimination, mm	5	5
**Temperature testing**		
Cold detection threshold, °C	32.1	31.5
Warm detection threshold,°C	44.6	39.8
Cold pain threshold, °C	0	4.2
Heat pain threshold, °C	44.4	43.5

*VAS, visual analogue scale; mN, Millinewton*.

## Discussion

Finding the cause of trigeminal sensory neuropathy can be a diagnostic challenge because it can be caused by a variety of disorders. The main causes of paresthesia in the maxillofacial region are dental in origin (2), whereas other systemic causes include demyelinating diseases, connective tissue diseases, systemic infection, and primary or metastatic malignancies, and can even be the first manifestation of multiple sclerosis (5). Forty-eight percent of dental causes have been attributed to a dental procedure and involve the IAN and lingual nerves (2). In this case, dental causes were excluded by clinical and radiological examination. A differential diagnosis was made between sickle cell disease (SCD), thalassemia minor, and HNPP as the possible cause of the idiopathic bilateral facial paresthesia. Other neurological causes were excluded by the Department of Neurology.

SCD is a group of hematological disorders that cause sickle-shaped erythrocytes to disrupt the blood flow in small vessels (6). This can lead to an acute vaso-occlusive sickle-cell crisis, characterized by tissue ischemia, inflammation, and periodic episodes of pain. Clinically evident peripheral nervous system involvement is uncommon in SCD patients. However, subclinical peripheral nerve association has been described (7). Gadallah et al. (8) also concluded that trigeminal neuropathy may be associated with SCD, either subclinically or symptomatically. Few case reports (9, 10) have described a relationship between SCDs and mental nerve neuropathy. Most reported cases had a homozygous (HbSS) or compound heterozygous (HbSC) form of the disease. The patients presented with bone pain crises at the mandible, followed by a burning sensation and numbness of the lower lip. It is thought that a vaso-occlusive sickle cell crisis may cause painful neuropathy due to nerve ischemia, infarction, or from compression as a result of bone infarct or osteomyelitis of the mandible (8). The patient in this case report was known to have the sickle cell trait, which is strictly not a form of SCD (6). He did not encounter the bone pain crises that often precede paresthesia or numbness. Thus, SCD was excluded as a cause of his symptoms.

Alpha-thalassemia is an inherited hemoglobinopathy characterized by impaired synthesis of alpha-globin chains, leading to excess beta-globin chains (11). This deficient production most frequently results from a deletion of one or more alleles (HBA1 and HBA2). The clinical presentation can vary depending on the number of affected alleles. Thus, alpha-thalassemia can be classified into different subtypes: silent alpha-thalassemia, alpha-thalassemia minor (or alpha-thalassemia trait), hemoglobin H disease (HbH), and hemoglobin Bart's hydrops fetalis. Alpha-thalassemia minor, the subtype in this case, is caused by a two-gene deletion, one in each chromosome, which causes microcytosis and hypochromia with absent or mild anemia, generally with no other symptoms. To the best of our knowledge, there are no records describing a correlation between alpha-thalassemia and peripheral neuropathy. In contrast to alpha-thalassemia, limited studies have (12, 13) reported an association between beta-thalassemia and sensory axonal polyneuropathy affecting the distal segments of the nerves. This phenomenon seems to increase with age, possibly be due to chronic anemia (13). Other hypothetical causes of this neuropathy are iron overload, neurotoxicity of the drug desferriosamine, and bone marrow expansion (14). The patient in this case did not present with any of the aforementioned hypothetical causes of neuropathy in beta-thalassemia. He was diagnosed with alpha-thalassemia minor, which has no proven association with peripheral neuropathy. Therefore, alpha-thalassemia minor was ruled out as a cause of the symptoms.

HNPP is an autosomal dominant disorder that affects the peripheral nerves and is characterized by recurrent episodes of transient mononeuropathy, usually provoked by minor trauma (15). It has a prevalence of 7–16 per 100,000 individuals and is frequently underdiagnosed due to the heterogeneity of the clinical and electrophysiological presentation (16). HNPP is caused genetically by a 1.5 Mb heterozygous deletion of the chromosome 17p11.2-p12 region, including the peripheral myelin protein-22 gene in the majority of cases. Symptoms usually start during adolescence or young adulthood and include episodes of numbness, paresthesia, muscle weakness, and atrophy. The neuropathological presentation includes segmental de- and re-myelination, tomaculous or sausage-like formations with typical segmental thickening of myelin, and diminished conduction velocity and amplitude of the potentials along motor and sensory nerves (17, 18). Electrodiagnostic, histopathological, and genetic testing are essential in diagnosing HNPP (15). The most frequently affected nerves are the median, ulnar, radial, and peroneal nerves, or the brachial plexus at sites commonly exposed to trauma or entrapment due to their anatomical locations (17). Cranial nerves are usually not involved. Only a few case reports (19–24) have described the involvement of the facial, hypoglossal, and other cranial nerves (Table 2).

**Table 2:** Case reports in the literature describing cranial nerve involvement in patients with HNPP.

**Study**	**Sex**	**Age (years)**	**Family history**	**Other/previous episodes**	**Cranial nerve involvement**	**Symptoms**	**Causative incident**	**Confirmed by molecular genetic analysis**	**Follow-up**
Corwin and Girardet (21)	Male	74	None	4 episodes of right foot drop following minor lower extremity trauma	Hypoglossal nerve	Dysarthria, inability to protrude or move the tongue laterally, left tongue fasciculations	Bilateral carotid endarterectomy	Yes	5 years: remaining sensation of swelling of the left tongue, atrophy of the right tongue, curling of the tongue tip to the left
Poloni et al. (19)	Male	16	Father diagnosed with HNPP	Left inferior peripheral facial palsy, complete right facial palsy, left peroneal deficit, transient right radial deficit	Facial nerve	Facial palsy	/	Yes	All episodes recovered in a few weeks
Ohkoshi et al. (23)	Female	19	Father and brother diagnosed with HNPP	2 episodes of hand drop, with bilateral severe weakness of wrist extensor muscles and finger extensor muscles and mild paresthesia on the radial side of both hands and forearms	Recurrent laryngeal nerve	Aphonia and hoarseness, dysphagia, aspiration of food and drink into the trachea	Sleeping in the prone position the night before	Yes	Complete recovery after 6 weeks, both hand drops remained
Iwasaki et al. (20)	Female	40	None	5-year history of increasing weakness in the lower extremities and deteriorating gait due to sensation of heaviness in her legs	CNS (trigeminal, facial, and hypoglossal nerves)	Dysesthesia in the left forehead, weakness of bilateral facial muscles, no protrusion of the tongue possible	/	Yes	/
Winter and Juel (22)	Female	19	Mother diagnosed with Charcot-Marie-Tooth disease	None	Hypoglossal nerve	Dysphagia, lingual dysarthria, tongue weakness, tongue deviation to the right with protrusion	Sleeping in a seated position, supporting the head with her right palm	Yes	Complete recovery in 10 days
Lorenzoni et al. (24)	Male	41	Sister and nephew similar symptoms, no proven HNPP	Recurrent episodes of mononeuropathies affecting mainly the ulnar, radial and peroneal nerves followed by spontaneous improvement since the age of 12 years	Recurrent laryngeal nerve, hypoglossal nerve	Swallowing dysfunction	None	Yes	Spontaneous improvement after few weeks, but with residual dysfunction when drinking liquid

Swallowing dysfunction and vocal cord paralysis have been described in HNPP in relation to hypoglossal neuropathy or recurrent laryngeal nerve palsy (21–23). Recurrent facial palsy was described as a first clinical manifestation in a family diagnosed with HNPP (19). The anatomy of the facial nerve leads to physiological entrapment sites, particularly in its intra-temporal portion, and makes it vulnerable to pressure palsies (20). To the best of our knowledge, facial paresthesia in HNPP has not been reported previously. These patients are predisposed to nerve demyelination, causing the slightest pressure, stretch, or repetitive movement to induce neurological impingement. This patient was previously diagnosed with HNPP, which was confirmed by genetic screening. He sometimes experienced recurrent episodes of muscle weakness and hypoesthesia in the arms and legs. As episodes of paresthesia are a symptom of HNPP and it is a slow progressive disorder, this facial paresthesia could be part of further progression of the disease. In this case, chewing and long conversations, which are repeated movements, could be a causative factor. Furthermore, QST and MRN showed changes in the trigeminal nerve, which could indicate a small fiber neuropathy. Currently, no treatment is available for HNPP (15). Patients usually recover from their symptoms, though it can sometimes take several months. Current management focuses mainly on preventing damage to the peripheral nerves. Activities that may provoke pressure palsies, such as prolonged sitting with crossed legs, repetitive movements of the wrist, and prolonged leaning on elbows, should be avoided.

## Conclusion

Clinical diagnosis and management of patients with spontaneous facial paresthesia remains a challenge for maxillofacial surgeons. Etiology can differ, but HNPP has never been reported among the known possible causes. After exclusion of local or iatrogenic etiologies, systemic or neurological causes should be considered. A good clinical examination and objective tests and imaging are imperative in obtaining an accurate diagnosis. Even though the presentation is rare, physicians should consider HNPP in the differential diagnosis of transient idiopathic trigeminal mononeuropathy, especially in the context of recurrent pressure palsies, distal extremity weakness, reduced tendon reflexes, or family history of hereditary neuropathy.

## Data Availability Statement

The raw data supporting the conclusions of this article will be made available by the authors, without undue reservation.

## Ethics Statement

Ethical review and approval was not required for the study on human participants in accordance with the local legislation and institutional requirements. The patients/participants provided their written informed consent to participate in this study. Written informed consent was obtained from the individual(s) for the publication of any potentially identifiable images or data included in this article.

## Author Contributions

LD wrote the manuscript with contributions of FV and LG. FV treated the patient. CP supervised the project. All authors contributed to manuscript revision, read, and approved the submitted version.

## Conflict of Interest

The authors declare that the research was conducted in the absence of any commercial or financial relationships that could be construed as a potential conflict of interest.

## Publisher's Note

All claims expressed in this article are solely those of the authors and do not necessarily represent those of their affiliated organizations, or those of the publisher, the editors and the reviewers. Any product that may be evaluated in this article, or claim that may be made by its manufacturer, is not guaranteed or endorsed by the publisher.
