# Supplementary material for: Facial Paresthesia, a Rare Manifestation of Hereditary Neuropathy With Liability to Pressure Palsies: A Case Report
Source: Front Neurol. 2021 Nov 16;12:726437. doi: 10.3389/fneur.2021.726437 (PMC8635109; doi:10.3389/fneur.2021.726437)
Supplement: Supplementary file 1 [file Data_Sheet_1.docx]

**Facial paresthesia, a rare manifestation of hereditary neuropathy with liability to pressure palsies: a case report.**

**Timeline.**

Assessment by the neurology department: Normal clinical examination.

02/2020

02/2020-

05-2020

Other diagnostic methods:

- Panoramic radiograph and lateral head film

- Blood analysis

- EMG of the left ulnar and median nerve

- Quantitative sensory testing

- Magnetic resonance neurography (MRN) of the trigeminal nerve

11/2019

Current illness: facial paresthesia, bilaterally in the distribution area of the mandibular nerve.

First presentation of bilateral facial paresthesia. The family doctor prescribed corticosteroids, without improvement of symptoms.

First consultation maxillofacial surgery:

Clinical examination: the trigeminal and facial nerves were normal.

11/2019

02/2021

09/2020

07/2020

Follow-up by phone: no recurrence or exacerbation of symptoms.

Consultation maxillofacial surgery: improvement of symptoms, reduction of number and intensity of episodes of facial paresthesia. Stop IM injections of vitamin B.

Consultation maxillofacial surgery: no improvement of symptoms. Start intramuscular injections of vitamin B once a week.

Final outcome for this episode of care: The episodes of facial paresthesia were decreased in number and intensity and did no longer impeded the patients’ daily life activities.

**Relevant past medical history:**

- heterozygote sickle cell trait

- hereditary neuropathy with liability to pressure palsies

- alpha-thalassemia minor

- tinnitus of unknown etiology

- second-degree atrioventricular block type 1
